# Beyond the Primary‐Secondary Dichotomy: Toward a Unified Metabolic Perspective

**DOI:** 10.1002/pei3.70225

**Published:** 2026-11-10

**Authors:** Davyson de Lima Moreira, Ygor Jessé Ramos, Renato Crespo Pereira

**Affiliations:** ^1^ Diretoria de Pesquisas Instituto de Pesquisas Jardim Botânico do Rio de Janeiro Rio de Janeiro Brazil; ^2^ Departamento do Medicamento, Faculdade de Farmácia Universidade Federal da Bahia Salvador Bahia Brazil; ^3^ Departamento de Biologia Marinha Instituto de Biologia, Universidade Federal do Rio de Janeiro Rio de Janeiro Brazil

**Keywords:** chemical ecology, metabolic engineering, metabolomics, plant–environment interactions, specialized metabolism, systems biology

## Abstract

The distinction between primary and secondary metabolism has long provided a useful conceptual and pedagogical framework in biochemistry, particularly in plant sciences, but it increasingly fails as a literal description of how metabolites are produced and function. Pathways share precursors, cofactors, regulatory mechanisms, transport systems, and recycling routes, while metabolites conventionally assigned to different categories can contribute to growth, signaling, defense, nutrient acquisition, structure, or stress responses depending on context. Here, we review the historical origins of the primary–secondary dichotomy and synthesize biochemical, genetic, ecological, metabolomic, and systems‐level evidence for a more integrated interpretation of plant metabolism. We do not argue that established terms must be abolished: they remain useful descriptors of broad tendencies in conservation, distribution, and pathway organization. Instead, we argue against treating them as fixed functional categories. We develop an operational framework in which network position, flux and resource coupling, phylogenetic distribution, spatiotemporal localization, conditional essentiality, and experimentally demonstrated function are evaluated together. We then show how this perspective changes the analysis of untargeted metabolomic data and the design of metabolic‐engineering strategies. A unified metabolic perspective provides a practical way to connect plant physiology, environmental responses, and ecological interactions without replacing established terminology with a new rigid vocabulary.

## 1 Introduction

The distinction between primary and secondary metabolism is deeply embedded in biochemistry, particularly in plant biology. In the nineteenth century, Julius Sachs described many plant compounds as metabolic “by‐products” that were formed but apparently not reused in the construction of new cells (Sachs 1873; Hartmann 2007). Albrecht Kossel subsequently formalized a distinction between primary and secondary cell constituents (Kossel 1891; Jones 1953). This historical framework proved convenient: metabolites associated with broadly conserved processes of growth and maintenance were grouped separately from the extraordinarily diverse compounds that later became associated with ecological interactions.

The functional interpretation of that division, however, was challenged early. Stahl's experiments on chemical protection against herbivores predated the formal primary/secondary terminology and anticipated a central principle of chemical ecology (Stahl 1888; Hartmann 2007). Fraenkel (1959) later argued that the “secondary” substances of plants were not metabolic waste, but adaptive chemicals shaped by interactions with other organisms. Subsequent research established specialized metabolites as major contributors to defense, signaling, mutualisms, stress tolerance, and plant fitness (Agrawal et al. 2015; Kessler and Kalske 2018; Müller and Junker 2022; Bai et al. 2024).

The challenge now extends beyond whether secondary metabolites are biologically important. Genetic and biochemical studies show that metabolites historically assigned to specialized metabolism can regulate growth and development, contribute substrates to broadly conserved pathways, and influence nutrient acquisition or cellular homeostasis (Erb and Kliebenstein 2020). Conversely, metabolites usually assigned to central metabolism can act as regulatory signals, extracellular cues, precursors of defensive chemistry, or mediators of plant–environment interactions; sucrose, malate, fatty acids, and glutamate provide well‐characterized examples (Rolland et al. 2006; Rudrappa et al. 2008; Wasternack and Feussner 2018; Liao et al. 2022). Primary and specialized metabolism are also coupled through shared carbon and nitrogen skeletons, energy, redox cofactors, subcellular compartmentation, transport, and feedback regulation (Maeda and Fernie 2021; Wang et al. 2022). Recent analyses of non‐canonical metabolic fluxes further emphasize that plant metabolic networks can redistribute flux through routes that are poorly represented by rigid pathway diagrams (Sweetlove et al. 2025).

This Review therefore addresses a narrower and more operational question than simply asking whether “secondary metabolism” exists. Haslam (1986) questioned the conceptual reality of the primary/secondary separation; Hartmann (2007) traced the historical transition from metabolic waste to ecologically relevant chemistry; Erb and Kliebenstein (2020) documented a blurred functional trichotomy among primary metabolites, secondary metabolites, and hormones; and Wang et al. (2022) emphasized the dynamic interface between primary and specialized metabolism. Building on these foundations, we ask how a unified metabolic perspective can be used in practice. Our contribution is to connect the historical and conceptual debate to an explicit interpretive framework for metabolomics, plant–environment studies, and metabolic engineering, while retaining established terminology as heuristic descriptors rather than fixed assignments of biological importance or function.

Importantly, a unified perspective does not imply that all metabolites are equivalent or that conventional categories contain no biological information. Metabolites classed as primary or central tend to be more broadly conserved and are often indispensable under standard growth conditions, whereas many specialized metabolites are lineage restricted, developmentally regulated, or strongly responsive to environmental context (Pichersky and Lewinsohn 2011; Weng et al. 2021; Wang et al. 2022). These are useful empirical tendencies. The problem arises when category membership is allowed to substitute for evidence about what a metabolite does in a particular organism, tissue, developmental stage, or environment.

## 2 Scope and Literature Selection

This article is a critical narrative Review rather than a systematic review or meta‐analysis. Literature was selected to represent four complementary evidence classes: (i) landmark historical sources that shaped the primary/secondary distinction; (ii) recent syntheses, particularly from the past decade, addressing metabolic integration, specialized metabolism, metabolomics, and metabolic engineering; (iii) mechanistic studies in which genetic, biochemical, physiological, or chemical‐complementation evidence demonstrates functions that cross conventional metabolic categories; and (iv) comparative examples that clarify the generality or limits of the argument. Historical statements were retained only when they could be traced to original sources or authoritative historical syntheses.

Greater weight was given to studies demonstrating in planta function, pathway coupling, or network consequences than to studies assigning function solely from compound class, abundance, or in vitro bioactivity. Counterexamples were accommodated by retaining genuine statistical differences between central and specialized metabolism, such as conservation and lineage specificity, while rejecting their interpretation as fixed functional hierarchies. Because Plant‐Environment Interactions is plant focused, terrestrial plant evidence forms the core of the Review. A small number of marine photosynthetic examples are retained only where they provide a particularly clear comparative demonstration of context‐dependent metabolic function. No formal PRISMA‐style inclusion process was applied because the purpose is conceptual synthesis rather than exhaustive evidence mapping; accordingly, we make no quantitative or bibliometric claims about the relative prevalence or impact of research on primary versus specialized metabolism.

## 3 From Historical Classification to a Metabolic Interface

The historical development of plant metabolic terminology reflects more than a succession of labels. It documents a progressive change in how the biological significance of metabolites has been interpreted, moving from classification based largely on presumed physiological necessity toward increasingly integrated views of metabolic function, evolution, and network organization (Figure 1). This progression is important because many of the assumptions embedded in the primary–secondary distinction originated under physiological and analytical conditions very different from those available today.

**FIGURE 1:**
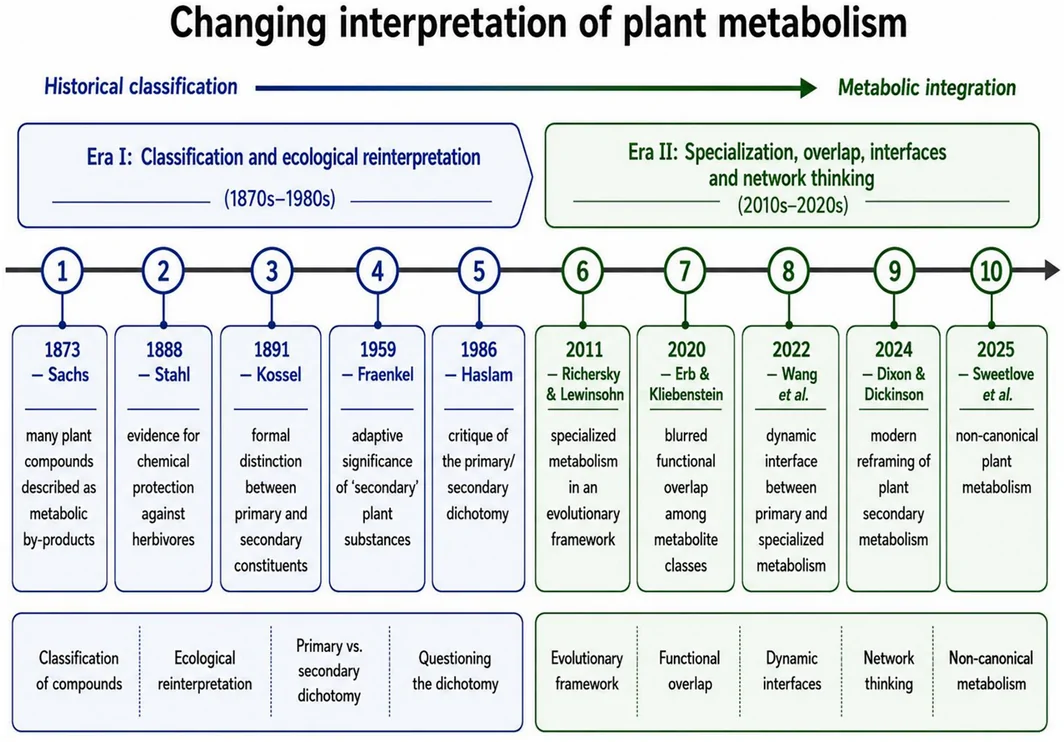
Selected milestones in the changing interpretation of plant metabolism. The timeline traces the shift from nineteenth‐century metabolic by‐products and primary/secondary classification toward ecological function, critique of rigid categories, functional overlap, and network‐based views of metabolic flux (Sachs 1873; Stahl 1888; Kossel 1891; Fraenkel 1959; Haslam 1986; Pichersky and Lewinsohn 2011; Erb and Kliebenstein 2020; Wang et al. 2022; Dixon and Dickinson 2024; Sweetlove et al. 2025).

In the nineteenth century, Sachs described numerous plant compounds as metabolic “by‐products”, reflecting the contemporary view that substances not directly reinvested in cell construction occupied a peripheral position in plant physiology (Sachs 1873; Hartmann 2007). Kossel subsequently formalized the distinction between primary and secondary cellular constituents (Kossel 1891; Jones 1953). Yet ecological observations were already complicating this interpretation. Stahl (1888) demonstrated that plant chemicals could protect against herbivores, anticipating the recognition that compounds apparently peripheral to growth could have major consequences for survival and fitness. Fraenkel (1959) transformed this observation into an explicitly evolutionary argument, proposing that many “secondary” plant substances were adaptive products shaped by interactions with herbivores rather than metabolic waste.

The next conceptual transition involved questioning not merely the presumed unimportance of secondary metabolites, but the validity of the boundary itself. Haslam (1986) argued that the separation between primary and secondary metabolism was to a considerable extent artificial, drawing attention to biosynthetic continuity and to the difficulty of assigning many compounds unambiguously to either category. This critique became increasingly relevant as biochemical and molecular approaches revealed that pathways conventionally placed in different metabolic domains share precursors, enzymes, cofactors, regulatory mechanisms, and cellular compartments.

During the genomic and evolutionary era, the increasing use of the term “specialized metabolism” represented an attempt to move away from the hierarchical implications of “secondary metabolism”. The concept also emphasized an important evolutionary property of plant chemistry: many specialized pathways are lineage restricted and arise through duplication, recruitment, and functional divergence of enzymes associated with more broadly conserved metabolism (Moghe and Last 2015; Pichersky and Lewinsohn 2011; Maeda and Fernie 2021; Weng et al. 2021; Ji et al. 2024). Specialized metabolism is therefore not external to central metabolism; it emerges through evolutionary modification of pre‐existing metabolic networks.

Recent work has pushed the discussion beyond terminology toward the properties of the metabolic system itself. Erb and Kliebenstein (2020) demonstrated extensive functional overlap among metabolites conventionally classified as primary, secondary, or hormonal, whereas Wang et al. (2022) emphasized natural variation at the interface between primary and specialized metabolism. Dixon and Dickinson (2024) further highlighted how compounds historically assigned to secondary metabolism participate in plant development, structure, regulation, and environmental responses. More recently, the recognition of non‐canonical metabolic fluxes has reinforced the idea that pathway diagrams represent predominant routes rather than immutable metabolic compartments, with flux being redistributed according to genotype, tissue, developmental state, and environmental conditions (Sweetlove et al. 2025).

These developments reveal a gradual conceptual transition from classification, through ecological reinterpretation and evolutionary specialization, toward functional overlap and network integration (Figure 1). The critical issue is therefore no longer simply whether “secondary” should be replaced by “specialized”. Either term can remain useful when it describes specific evolutionary or biochemical tendencies, but the problem arises when categorical membership is allowed to predetermine biological significance. A more informative approach is to move from classification‐first reasoning to evidence‐first reasoning, evaluating a metabolite through its network position, flux, phylogenetic distribution, spatiotemporal localization, conditional importance, and experimentally demonstrated functions.

## 4 Why the Boundaries Are Biochemically and Functionally Porous

### 4.1 Biosynthetic Continuity and Network Architecture

Plant metabolic pathways are coupled at multiple levels. Carbon fixed through photosynthesis and intermediates generated by glycolysis, the tricarboxylic acid cycle, the pentose‐phosphate pathway, and amino‐acid metabolism provide precursors for phenylpropanoids, terpenoids, alkaloids, glucosinolates, and many other specialized products (Caretto et al. 2015; Maeda and Fernie 2021; Méteignier et al. 2023). These branches draw on common pools of carbon, nitrogen, ATP, reducing equivalents, and enzyme capacity, making their regulation dependent on the state of the wider metabolic network (Caretto et al. 2015; Maeda and Fernie 2021; Wang et al. 2022).

The connection is also spatial. Specialized pathways frequently span organelles, cell types, tissues, or organs, requiring transport of intermediates and coordination among distinct metabolic compartments (Jacobowitz and Weng 2020; Lacchini and Goossens 2020; Kanstrup and Nour‐Eldin 2022; Shen et al. 2024). Compartmentation can separate toxic intermediates, concentrate substrates, or create tissue‐specific functions, but it also creates dependencies on transport and exchange. Spatial separation is therefore an additional layer of metabolic integration rather than evidence that a pathway operates independently from central metabolism.

Finally, metabolic networks can support alternative flux distributions across tissues and environmental conditions. Stable‐isotope approaches, metabolic flux analysis, and computational models show that canonical pathway diagrams represent common routes rather than the only possible solutions (Shih and Morgan 2020; Sweetlove et al. 2025). This flexibility becomes particularly important when environmental stress, developmental change, or genetic engineering alters demand for carbon skeletons, energy, reducing power, or storage capacity.

### 4.2 Metabolites Can Cross Conventional Functional Categories

The strongest challenge to a rigid dichotomy comes from metabolites whose experimentally demonstrated functions cross the expected boundary. In *Arabidopsis thaliana* , glucosinolate‐derived compounds influence stomatal closure, while an evolutionarily young glucosinolate modulates root growth through the conserved TOR signaling pathway (Hossain et al. 2013; Malinovsky et al. 2017). These observations do not make glucosinolates “primary metabolites”; they show that lineage‐associated defensive chemistry can become integrated into physiological regulation.

Benzoxazinoids provide another illustration. Mutants deficient in these compounds can display iron deficiency despite iron availability because benzoxazinoids participate in soil iron acquisition (Hu et al. 2018). Flavonoids, classically treated as specialized metabolites, influence PIN‐mediated auxin transport and can contribute carbon to the benzenoid moiety of the respiratory cofactor ubiquinone (Santelia et al. 2008; Soubeyrand et al. 2018). These are mechanistic connections between compounds conventionally assigned to specialized metabolism and processes central to growth, development, and cellular function.

The converse is equally important. Molecules usually assigned to primary or central metabolism frequently acquire signaling or ecological roles. Sucrose is both a carbon and energy source and a regulatory signal capable of reprogramming gene expression and specialized metabolic output (Rolland et al. 2006). Linoleic and linolenic acids are membrane and storage components but also feed oxylipin pathways that generate jasmonates, which coordinate defense, development, and stress responses (Wasternack and Feussner 2018). Malate released by *Arabidopsis thaliana* roots can recruit *Bacillus subtilis* and contribute to induced resistance (Rudrappa et al. 2008), while glutamate couples carbon–nitrogen metabolism with signaling involved in growth, wounding, and defense (Liao et al. 2022).

Terpenoids illustrate why even chemical‐class labels do not map cleanly onto metabolic categories. Widely conserved terpenoids participate in photosynthesis, electron transport, membrane organization, and hormone signaling, whereas lineage‐restricted terpenoids often mediate defense and communication. Both sets arise from shared isoprenoid precursor chemistry and are connected by crosstalk between the MVA and MEP pathways (Bergman and Dudareva 2024; Bergman et al. 2024). The same broad chemical family therefore spans functions traditionally placed on opposite sides of the primary/secondary divide.

A comparative example from brown seaweeds further illustrates context dependence. Phlorotannins are commonly discussed as defensive or stress‐responsive phenolics, yet phenolic compounds packaged in physodes are also secreted into zygote walls and cell plates and contribute to cell‐wall construction (Schoenwaelder and Clayton 1998; Gómez and Huovinen 2020). Brown seaweeds are not land plants, so this example is not used as primary evidence for plant metabolism. Its comparative value is that the same chemical family can contribute to ecological interactions and structural organization in another photosynthetic lineage, reinforcing the principle that function depends on localization and biological context.

### 4.3 Environmental Context Changes What a Metabolite Does

Plant metabolism is not only interconnected; it is conditional. Genotype, developmental stage, tissue identity, resource availability, and biotic or abiotic stress alter metabolic fluxes, spatial allocation, and the functions that become consequential for performance (Lacchini and Goossens 2020; Panda et al. 2021; Müller and Junker 2022). The chemical phenotype is therefore better viewed as a dynamic component of plant niche expression than as a static inventory of compounds.

This conditionality helps explain why “essential” and “nonessential” are poor universal descriptors. The contribution of a metabolite to plant performance may be minor under benign laboratory conditions but become substantial under herbivory, pathogen attack, resource limitation, water stress, high irradiance, or competition (Panda et al. 2021; Weng et al. 2021; Bai et al. 2024). Conversely, constitutive production of protective chemistry may carry costs when the relevant stress is absent. Experimental and synthetic‐biology approaches increasingly show that the fitness contribution of specialized metabolites depends on ecological setting, tissue, concentration, and interaction partner rather than on a universal ranking of metabolic importance (Kessler and Kalske 2018; Panda et al. 2021; Weng et al. 2021; Bai et al. 2024).

Environmental responses also redistribute resources across conventional pathway boundaries. Defense induction can alter carbon and nitrogen allocation, redox state, and growth, while environmental cues regulate specialized metabolism through transcriptional, post‐transcriptional, post‐translational, transport, and compartmentation mechanisms (Caretto et al. 2015; Lacchini and Goossens 2020; Méteignier et al. 2023). Conditional importance is therefore not equivalent to biological unimportance; it is a property of how metabolic function is expressed under specific particular circumstances.

## 5 A Unified Metabolic Perspective Without a New Dichotomy

The aim of a unified metabolic perspective is not to replace the primary/secondary distinction with a new binary vocabulary. Any new pair of labels would risk reproducing the same problem if category membership were again treated as a shortcut for biological importance or function. Instead, primary, secondary, specialized, central, and related terms are most useful when their intended meaning is made explicit for the question being asked.

For example, “central metabolism” can appropriately denote highly connected pathways that carry substantial flux and supply precursors; “specialized metabolism” can appropriately describe pathways with restricted phylogenetic distributions or lineage‐specific diversification (Pichersky and Lewinsohn 2011; Maeda and Fernie 2021; Weng et al. 2021; Ji et al. 2024). In ecological studies, however, neither term should predetermine whether a metabolite has structural, regulatory, defensive, signaling, or nutritional functions (Erb and Kliebenstein 2020; Weng et al. 2021). Classification and function answer different questions.

A practical alternative is to interpret metabolites along several complementary dimensions. Before inferring biological or ecological significance, investigators can ask where a metabolite sits in the network, how its production changes flux and resource allocation, how broadly its biosynthesis is conserved, where and when it accumulates, whether its loss is deleterious constitutively or only under specific conditions, and which functions have been experimentally demonstrated (Table 1). These dimensions are not intended to create a new classification system. They are prompts that prevent a historical label from substituting for mechanistic evidence.

**TABLE 1:** Operational questions for interpreting metabolites within a unified metabolic perspective.

Dimension	Question	Why it matters	Illustrative consequence
Network position	Which pathways produce, consume, or exchange the metabolite?	Reveals connectivity and prevents a biosynthetic branch from being treated as isolated	A phenylpropanoid increase may depend on changes in upstream carbon and aromatic amino‐acid metabolism (Caretto et al. 2015; Wang et al. 2022)
Flux and resource coupling	How does production change carbon, nitrogen, ATP, reducing power, and competing sinks?	Distinguishes changes in pool size from changes in pathway throughput and resource allocation	A larger metabolite pool does not necessarily indicate greater biosynthetic flux, because pool size also reflects consumption, turnover, and competing fluxes (Shih and Morgan 2020)
Phylogenetic distribution	Is the pathway broadly conserved or lineage restricted?	Captures genuine evolutionary information without equating restricted distribution with low importance	A lineage‐specific metabolite can nevertheless make a major contribution to fitness in the ecological context in which it evolved (Weng et al. 2021; Bai et al. 2024)
Spatiotemporal localization	Where and when are the metabolite and pathway components present?	Function depends on tissue, cell type, organelle, developmental stage, and environmental state	The same chemical family may contribute to distinct functions depending on its subcellular, cellular, tissue, or developmental localization (Jacobowitz and Weng 2020; Lacchini and Goossens 2020; Weng et al. 2021)
Conditional essentiality	What happens when biosynthesis is disrupted under benign and challenging conditions?	Separates universal necessity from context‐dependent fitness effects	A biosynthetic mutant with little phenotype under benign conditions may show reduced performance when exposed to nutrient limitation, herbivory, or other environmental challenges (Weng et al. 2021; Bai et al. 2024)
Demonstrated function	What role is supported by genetic, physiological, biochemical, or ecological evidence?	Prevents function from being inferred from conventional category, abundance, or in vitro activity alone	Metabolic labels should be treated as descriptive metadata; functional claims require genetic, physiological, biochemical, or ecological evidence (Weng et al. 2021; Bai et al. 2024)

This operational emphasis is where the present perspective extends earlier critiques. Haslam (1986) established the conceptual problem; Hartmann (2007) documented the historical and functional reappraisal of secondary metabolism; Erb and Kliebenstein (2020) demonstrated extensive functional overlap; and Wang et al. (2022) described a dynamic interface between primary and specialized metabolism. The approach proposed here builds on those insights to guide metabolite interpretation in experiments, comparative studies, metabolomics, and metabolic engineering. At the system level, this perspective is represented as an integrated, context‐dependent metabolic network in which carbon and nitrogen metabolism, energy and redox processes, biosynthetic branches, transport, compartmentation, turnover, and recycling are dynamically connected and jointly shaped by developmental and environmental conditions (Figure 2).

**FIGURE 2:**
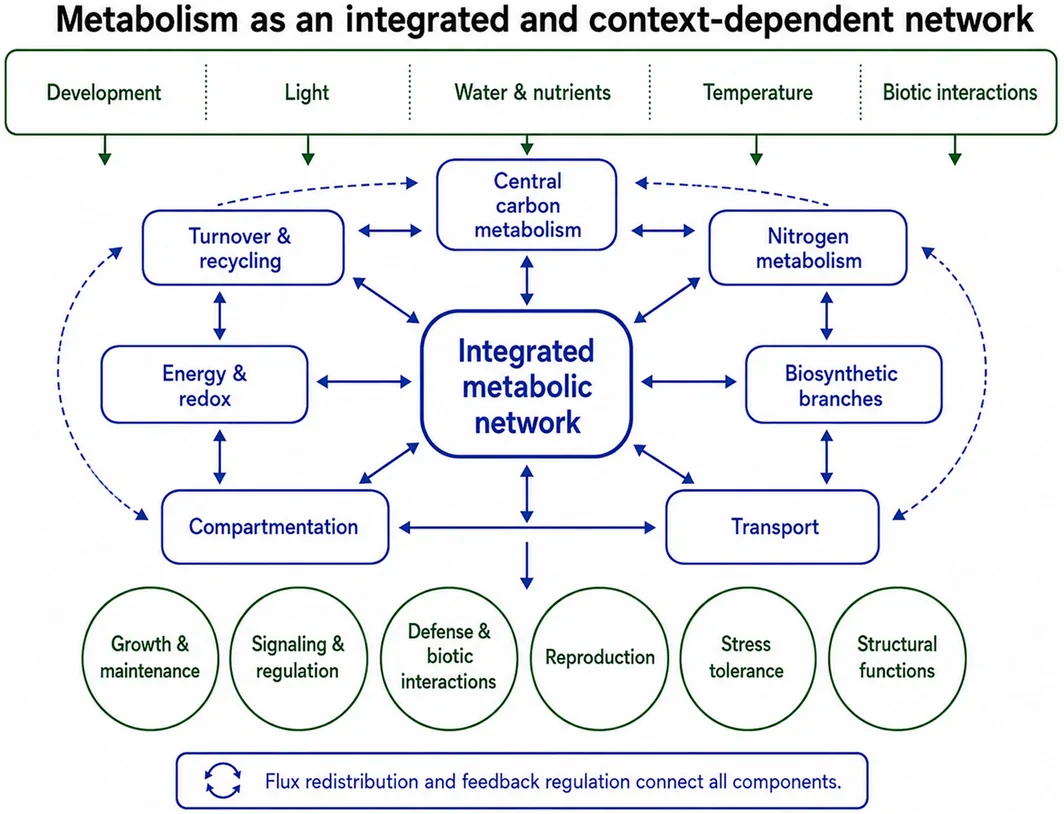
Metabolism as an integrated, context‐dependent network. Carbon, nitrogen, energy/redox metabolism, biosynthetic branches, transport, compartmentation, turnover, and recycling are linked by bidirectional flux and regulation. Developmental and environmental cues act across this network, generating overlapping outputs in growth, structure, signaling, defense, reproduction, and stress tolerance.

## 6 What Changes in Untargeted Metabolomics?

Untargeted metabolomics is a natural testing ground for a unified metabolic perspective because analytical platforms detect chemical features before biological categories are assigned to them. High‐resolution mass spectrometry can recover broad portions of plant chemical diversity, yet annotation remains incomplete, and many detected features cannot be confidently named (Li and Gaquerel 2021; Alseekh and Fernie 2023). Forcing these features into “primary” or “specialized” bins before examining covariance, pathway context, localization, or phenotype association can therefore introduce assumptions that are not present in the data.

Under a conventional split workflow, amino acids, sugars, and organic acids may be analyzed primarily as physiological variables, whereas phenolics, terpenoids, or alkaloids are analyzed separately as defensive or stress‐related chemistry. A unified workflow changes the order of inference. First, features are detected and annotated with explicit confidence levels. Second, metabolites are mapped onto biochemical networks and co‐variation modules without requiring a primary/secondary boundary. Third, changes are related to physiological traits, environmental treatments, transcript or protein abundance, spatial information, and, when possible, flux measurements. Functional labels are assigned only after these relationships are evaluated and may remain multiple or provisional.

A worked analytical workflow can be illustrated by an experiment in which drought and herbivory alter sugars, amino acids, malate, oxylipins, phenylpropanoids, and glucosinolate‐related features in the same plants. A category‐first interpretation could generate two parallel narratives: “primary metabolism” is reprogrammed by resource stress, whereas “secondary metabolism” mounts defense. The unified interpretation instead tests whether the pattern reflects coordinated carbon–nitrogen reallocation, redox adjustment, signaling, and biosynthetic demand across one network. Co‐variation modules can then be related to water status, growth, herbivore resistance, and recovery, while isotope tracing or transcript integration can distinguish changes in pool size from changes in flux. The chemical classes remain informative, but they no longer determine the biological narrative in advance.

Empirical studies already illustrate the value of integrated analysis. Metabolomic profiling of *Brassica rapa subsp. Chinensis* has revealed coordinated variation across metabolites conventionally assigned to primary and secondary metabolism (Yeo et al. 2021), while multi‐omics approaches increasingly resolve natural variation at their interface (Wang et al. 2022). The framework also encourages caution: a feature associated with a stress treatment is not automatically a defense compound, and an unidentified feature should not be called “secondary” simply because it is absent from a central‐pathway database. Network association, isotope tracing, spatial metabolomics, genetics, and ecological validation can progressively strengthen functional inference (Jacobowitz and Weng 2020; Li and Gaquerel 2021; Shen et al. 2024).

## 7 Implications for Metabolic Engineering and Synthetic Biology

The same conceptual shift has direct consequences for metabolic engineering. Engineering a desired specialized product is often described as modification of a terminal pathway, but sustained high production requires coordinated provision of precursors, ATP, reducing power, cofactors, transport capacity, storage or secretion, and tolerance of pathway intermediates. These requirements couple the engineered branch to central metabolism and to cellular organization (Jacobowitz and Weng 2020; Shih and Morgan 2020; Bergman et al. 2024).

Flux‐based approaches make this dependence explicit. Metabolic flux analysis can identify competing sinks and test whether increasing the expression of a pathway enzyme increases carbon flow toward the target product (Shih and Morgan 2020). Wang et al. (2022) similarly identified the primary–specialized interface as a major target for precise metabolic modification. More recent work shows that cell‐type specificity, subcellular compartmentation, transport, and post‐translational control can strongly affect engineering outcomes (Kanstrup and Nour‐Eldin 2022; Ntelkis et al. 2024; Shen et al. 2024).

A unified perspective therefore changes the engineering question from “How can the specialized pathway be increased?” to “How can the network that supports the desired flux be reconfigured without destabilizing the rest of metabolism?” Sweetlove et al. (2025) make this principle especially clear for high‐flux products: enhanced output may depend on non‐canonical supporting fluxes in central metabolism that supply carbon skeletons, energy, and reducing equivalents. Synthetic biology extends the same logic by allowing pathway localization, transport, externalization, and metabolite diversity to be manipulated as functional properties rather than treating final product titre as the only endpoint (Bai et al. 2024).

This network view can also reduce unintended trade‐offs. Increasing precursor withdrawal into one branch may compromise growth, reproduction, membrane synthesis, or stress responses; conversely, increasing precursor supply without addressing storage, transport, enzyme balance, or feedback inhibition may fail to increase yield. Metabolic engineering is therefore an applied demonstration of the Review's central argument: pathway labels are less informative than the fluxes, constraints, and regulatory connections that join them.

## 8 Consequences for Plant–Environment Interactions

For Plant‐Environment Interactions, the value of a unified metabolic perspective is that it connects environmental responses to the full chemical phenotype of the plant. Herbivory, pathogens, nutrient availability, drought, salinity, light, and temperature do not perturb an isolated “secondary” compartment; they alter resource acquisition, energy balance, signaling, growth, and interaction chemistry simultaneously. The metabolic phenotype that emerges is therefore a coordinated response of an interconnected network (Kessler and Kalske 2018; Panda et al. 2021; Méteignier et al. 2023).

This view also discourages an artificial separation between physiology and chemical ecology. A compound that contributes to defense may simultaneously regulate development or nutrient status, while a metabolite conventionally assigned to central metabolism may shape interactions through exudation or signaling. Ecological consequences depend on the receiver, metabolite concentration, localization, timing, and environmental context (Kessler and Kalske 2018; Lacchini and Goossens 2020; Müller and Junker 2022). Treating metabolites as components of a dynamic chemical phenotype is therefore more compatible with contemporary trait‐ and niche‐based ecology than assuming that ecological function belongs to one metabolic category (Müller and Junker 2022).

Future plant–environment studies can test this integration directly. Designs that combine untargeted metabolomics with physiological measurements, isotope tracing, spatial or single‐cell approaches, transcriptomics, and ecological performance can determine how environmental change redistributes metabolic flux and alters function across levels. Such approaches will also help distinguish correlation from function, a persistent limitation of metabolomics‐based ecological inference (Li and Gaquerel 2021; Bai et al. 2024; Shen et al. 2024).

## 9 Conclusion

The primary/secondary distinction has substantial historical and heuristic value, but it should no longer be treated as a fixed map of metabolic function or biological importance. Plant metabolism is a network in which shared precursors, cofactors, fluxes, compartmentation, transport, turnover, and regulatory feedback connect pathways traditionally assigned to different categories. Experimental evidence further shows that metabolites can perform structural, regulatory, physiological, and ecological roles that cross conventional boundaries.

A unified metabolic perspective does not require familiar terminology to be abandoned. It requires greater precision about what that terminology is being used to describe. “Primary,” “central,” “secondary,” and “specialized” can remain useful descriptors of broad patterns in conservation, network position, flux, or lineage specificity, if they are not used as substitutes for functional evidence. In practice, metabolite interpretation should integrate network position, flux and resource coupling, phylogenetic distribution, spatiotemporal localization, conditional essentiality, and experimentally demonstrated function.

The conceptual gain becomes most visible in application. In metabolomics, the approach favors network‐ and phenotype‐centered interpretation before categorical assignment. In metabolic engineering, it redirects attention from isolated terminal pathways toward precursor supply, flux redistribution, redox and energy balance, compartmentation, transport, and feedback. In plant–environment research, it links physiology and chemical ecology as different manifestations of the same dynamic metabolic system.

The next generation of isotope‐resolved metabolomics, spatial metabolomics, single‐cell approaches, multi‐omics integration, and functional genetics should make these connections increasingly testable. The enduring question is therefore not whether a compound is primary or secondary, but how its production, localization, flux, and function contribute to plant performance in a particular biological and environmental context.

## Funding

The authors have nothing to report.

## Disclosure

The authors have nothing to report.

## Conflicts of Interest

The authors declare no conflicts of interest.

## Data Availability

No new data were generated or analyzed in support of this review.
